# Cytolytic T lymphocytes from HLA-B8^+ ^donors frequently recognize the Hodgkin's lymphoma associated latent membrane protein 2 of Epstein Barr virus

**DOI:** 10.1186/2042-4280-2-4

**Published:** 2011-02-11

**Authors:** Ming L Tsang, Christian Münz

**Affiliations:** 1 Laboratory of Cellular Physiology and Immunology, The Rockefeller University, New York, USA; 2 Viral Immunobiology, Institute of Experimental Immunology, University of Zürich, Zürich, Switzerland

## Abstract

Epstein Barr virus (EBV) is a lymphotrophic γ-herpesvirus that infects more than 90% of the adult human population. It transforms B cells in vitro and is associated with lymphomas in vivo. In most EBV carriers the emergence of these malignancies, however, is prevented by T cell mediated immune control. Part of this control is mediated by CD8^+ ^T cells, which mainly target a subset of viral nuclear antigens, EBNA3A, B and C, in healthy EBV carriers. In HLA-B8 positive individuals, the dominant CTL response is biased towards recognition of EBNA3A. However, spontaneously arising EBV-associated malignancies, such as Hodgkin's lymphoma and nasopharyngeal carcinoma do not express EBNA3s and instead express latent membrane protein 2 (LMP2) as well as LMP1 and EBNA1. Here we describe the new HLA-B8 restricted, LMP2 derived CD8^+ ^T cell epitope, LMP2_345-352_. Although the frequency of LMP2_345-352 _specific CD8^+ ^T cells is usually lower than immunodominant EBNA3A specific CD8^+ ^T cells in fresh blood, the former can be expanded in the majority of HLA-B8^+ ^EBV carriers after 1 week co-culture with peptide pulsed dendritic cells. We demonstrate that LMP2_345-352 _specific CD8^+ ^T cells secrete IFN-γ and kill both peptide pulsed targets as well as HLA-B8 matched LCL and LMP2 expressing Hodgkin's lymphoma cells. We suggest that cytotoxic CD8^+ ^T cell responses against LMP2 coexist with the immunodominant EBNA3 specific responses in healthy EBV carriers and help to resist EBV associated malignancies.

## Introduction

The human γ-herpesvirus Epstein Barr virus (EBV) establishes a life-long, mostly asymptomatic infection in more than 90% of human adults. In immune competent hosts, it persists in latently infected B cells. EBV reservoirs under immune control are located in the peripheral blood and secondary lymphoid organs like the tonsils. Virus infected naïve B cells in tonsils of healthy EBV carriers express all latent antigens, the six nuclear antigens (EBNA1, 2, 3A, 3B, 3C and LP) and the two membrane proteins (LMP1 and 2)[1]. This expression pattern can also be found after transformation of B cells in immunosuppressed hosts or in vitro generated lymphoblastoid cell lines (LCL)[2]. The subset of virus carrying centroblasts and centrocytes in donors that have established immune control of EBV only express EBNA1, LMP1 and LMP2[1,3]. This expression pattern is also found in most spontaneously arising EBV associated malignancies, like Hodgkin's disease and nasopharyngeal carcinoma[4]. In peripheral blood, EBV can be found in memory B cells expressing none of the latent EBV antigens[5] or EBNA1 during homeostatic proliferation[6]. Thus, healthy EBV carriers already harbor EBV infected B cells with transforming viral antigen expression patterns similar to EBV associated malignancies.

EBV transformed B cells are controlled by T cell immunity in healthy EBV carriers[7]. This becomes apparent in patients with T cell compromising co-infection with the human immunodeficiency virus (HIV), genetic lesions like X-linked lymphoproliferative disease (XLP) and immunosuppressive treatment after allograft transplantation. In all these instances EBV associated lymphomas occur at increased frequencies with especially in XLP patients often fatal outcome[4,8-10]. Some of these can be treated by adoptive transfer of in vitro expanded T cell lines[11], suggesting that T cells form a crucial component of EBV specific immune control.

Some of these lines are dominated by CD8^+ ^T cells specific for EBNA3 proteins[12,13]. Restricted by the common HLA-B8 haplotype, EBNA3A can be recognized with high frequency by CD8^+ ^T cells (EBNA3A_325-333_: 1-3% in acute infectious mononucleosis and 0.1-1% in healthy EBV carriers)[14], and forms the immunodominant specificity in this HLA background. However, EBNA3A, 3B and 3C are not expressed in most spontaneously arising EBV malignancies, like Hodgkin's disease and nasopharyngeal carcinoma, and other specificities are required to mediate resistance against these malignancies by most EBV^+ ^individuals. Of the antigens expressed in these tumors, i.e., EBNA1, LMP1 and LMP2, only LMP2 is frequently recognized by CD8^+ ^T cells[15-17]. Thus, we addressed if HLA-B8^+ ^healthy EBV carriers carry in addition to their immunodominant EBNA3A specific response LMP2 specific CD8^+ ^T cells, which might allow them to resist the development of EBV associated malignancies that lack EBNA3 expression. Indeed, we frequently found in HLA-B8^+ ^healthy donors CD8^+ ^T cells for the new LMP2_345-352 _epitope, although their frequency is usually lower than that of EBNA3A specific CD8^+ ^T cells in fresh blood. However, after 1 week expansion with peptide pulsed dendritic cells, the frequency of LMP2_345-352 _specific, HLA-B8 restricted CD8^+ ^T cells is similar to LMP2_426-434 _specific, HLA-A2 restricted and EBNA3A_325-333 _specific, HLA-B8 restricted CD8^+ ^T cells. LMP2_345-352 _specific CD8^+ ^T cell clones secrete IFN-γ and show cytolytic activity upon encounter of 10 nM peptide pulsed targets, HLA-B8 matched LCL and HLA-B8^+ ^LMP2 expressing Hodgkin's cells. The LMP2 and EBNA3A specific CD8^+ ^T cell responses also can be visualized in fresh blood by staining for IFN-γ production and CD69 expression. While intracellular cytokine staining detected only a fraction of the stimulated T cells, CD69 staining leads to identification of nearly all antigen reactive cells. Our findings indicate that the presence of LMP2 specific CD8^+ ^T cells can be detected even in HLA-B8^+ ^individuals, whose latent EBV antigen specific CD8+ T cell response is usually dominated by EBNA3A specific cells. We suggest that LMP2 specific CD8^+ ^T cell responses contribute to protection against spontaneously arising EBV associated tumors in healthy carriers and should be exploited in cancer patients lacking this immune control.

## Materials and methods

### Cell lines

The EBV transformed B cell lines LG2 (HLA-B8^-^)[18], BC-LCL (HLA-B8^-^), CM-LCL (HLA-B8^-^), DC-LCL (HLA-B8^-^), SO-LCL (HLA-B8^+^), JT-LCL (HLA-B8^+^) and BM-LCL (HLA-B8^+^)[19] were cultured in RPMI-1640 + 10% FCS + glutamine + gentamicin. The CEM/LCL hybrid T2[20] and the EBV^- ^Hodgkin's lymphoma cell line HDLM-2 (DMSZ, Braunschweig, Germany) were also cultured in in RPMI-1640 + 10% FCS + glutamine + gentamicin.

### Dendritic cells (DC)

As previously described[21], PBMCs from leukocyte concentrates (buffy coats of the New York blood bank) were isolated on Ficoll-Paque (Pharmacia) and separated by rosetting into T-cell enriched (ER^+^) and depleted (ER^-^) populations. 3 × 10^6 ^ER^- ^PBMC/well or adherent cells from 10^7 ^PBMC/well were plated in 6-well plates with RPMI-1640 + 1% single donor plasma + glutamine + gentamicin. 500 μl medium were added at day 2, 4 and 6 per well. rhIL-4 and rhGM-CSF were added to a final concentration of 500 and 1000 U/ml, respectively, at day 0, 2, 4 and 6. On day 6, the floating immature DCs were transferred to new plates at 10^6 ^cells/well and half of the medium was replaced with fresh medium containing IL-1β/IL-6/TNFα/PGE_2 _(all except PGE2 from R&D Systems, Minneapolis, MN; PGE2: Sigma, St. Louis, MO) to mature the DCs for two days. The maturation cytokines were added to a final concentration of IL-1β, 10 ng/ml, IL-6, 1000 U/ml, TNFα, 10 ng/ml, and PGE_2_, 1 μg/ml. DCs and T cells were used fresh or following cryopreservation. DCs were infected with recombinant vaccinia viruses (MOI 2) after maturation as described previously[19].

### Vaccinia virus stock generation and infection of DCs

Recombinant vaccinia viruses (vv) were expanded and titrated as previously described[19].

### Generation of CD8^+ ^T cell lines and clones

Positive selection for CD8^+ ^PBMC from serologically HLA-B8^+ ^typed leucocyte concentrates was performed using αCD8-MicroBeads, MS^+^/RS^+ ^columns and MiniMACS separator (Miltenyi Biotec, Bergisch-Gladbach, Germany). CD8^+ ^T cells were stimulated with irradiated (3000 rad) mature DCs at a ratio of 30:1 (T:DC) or irradiated (20000 rad) LCL at a ratio 10:1 (T:DC) in RPMI + 5% AB serum + glutamine + gentamicin. DCs were pulsed for one hour with synthetic peptides at 37°C and 10 μM peptide concentration in serum-free RPMI prior to stimulation. LMP2_345-352 _specific CD8^+ ^T cells were cloned at 10, 1 or 0.3 T cells/well in RPMI + 8% AB serum + 150 IU/ml rhIL-2 (Chiron, Emeryville, CA) + 1 μg/ml PHA-L (Sigma, St. Louis, MO) + glutamine + gentamicin. 10^5 ^irradiated PBMC (3000 rad) and 2 × 10^4 ^irradiated LCL (20000 rad)/well were added as feeders[22]. After 14d, expanding wells were tested in split-well IFN-γ ELISPOT assays against LMP2_345-352_, LMP2_347-355_, HLA-B8 matched and mismatched LCL.

### FACS analysis

T cell clones were stained with simultest αCD4-FITC/αCD8-PE and isotype controls IgG1-FITC/IgG2a-PE (BD Pharmingen, San Diego, CA). Samples were analyzed on a FACScalibur (BD Pharmingen). HLA-B8^+ ^leucocyte concentrate donors were identified by staining of PBMC with αHLA-B8 antibody (One Lambda, Canoga Park, CA) and αmouseIgM-PE (Biosource, Camarillo, CA).

### ELISPOT assay

ELISPOT assays were performed as previously described[21]. MAHA S45 plates (Millipore, Bedford, MA) were coated with αIFN-γ antibody 1-D1K (MABTECH). Plates were blocked with DMEM + 5% HS. Afterwards, indicated amounts of responder T cells and 3 × 10^3 ^stimulator DCs, 10^4 ^LCL, 10^4 ^Hodgkin's lymphoma cells or 1 pM to 25 μM of the respective peptide epitopes were added per well and incubated for 1 day. Where indicated target cells were pulsed with 10 μM of the respective peptides for 1 h at 37°C in RPMI without supplements and then washed before addition to the ELISPOT co-cultures. Then the plates were incubated with biotinylated αIFN-γ antibody 7-B6-1 (MABTECH). Afterwards, preassembled avidin-peroxidase-complexes Vectastain ABC kit (Vector laboratories, Burlingame, CA) was added. Spots were developed by addition of stable DAB (Research Genetics, Huntsville, AL). Plates were washed 3 times with water and air-dried. SFC (spot forming cells) were counted using a stereomicroscope (mean counts of triplicates). Where indicated, αHLA-DR antibody L243[23], αHLA-A, B, C antibody w6/32[24] or αHLA-B8 antibody (One Lambda) was added at the indicated concentrations.

### ^51^Cr release assay

Targets were labeled with 50 μCi Na_2_^51^CrO_4 _for 45 min at 37°C. Labeled targets were incubated for 4-5 h with CTL in RPMI + 10% FCS + 2 mM glutamine. Afterwards an aliquot of the supernatant was harvested and counted in Wallac OptiPhase SuperMix scintillation fluid in a Wallac 1450 MicroBeta TriLux plate counter (Wallac, Turku, Finland). Percent specific lysis was calculated by ([cpm experimental well - cpm spontaneous release]/[cpm maximum release - cpm spontaneous release]) × 100%. Spontaneous release was determined by incubating the labeled targets with medium, and maximum release by incubating targets in 1% Triton X-100 solution.

### Intracellular cytokine assay

500 μl of heparinized whole blood was placed in 5 ml polypropylene tubes (Fischer Scientific, Pittsburgh, PA) with 1 μg/ml of each of anti-CD28 and anti-CD49d (BD Pharmingen), 10 μg/ml Brefeldin A (Sigma) and either RPMI (negative control), 10 μg/ml PHA (positive control), 10 μl 1 mM LMP2_345-352_, LMP2_347-355 _and EBNA3A_325-333 _solution. Cells were incubated for 6 hours at 37°C in 5% CO_2_. 20 mM EDTA was added for 15 minutes followed by 9 volumes of FACS Lysing Solution (BD Pharmingen) for 10 minutes at RT. Samples were then frozen at -80°C. Samples were subsequently thawed and permeabilized with 0.1% saponin and 0.1% BSA for ten minutes at RT. Cells were spun down and supernatant was decanted. The αIFN-γ-PE, αCD8-PerCP and αCD69-FITC antibodies (BD Pharmingen) were added for 30 minutes at room temperature. Samples were then washed with the permeabilization solution and fixed with 1% PFA. FACS acquisition was performed on a 4 color FACSsort (BD Pharmingen). Gates were set on CD8^+ ^cells, and autofluorescent cells were excluded in a channel for which no fluorochrome was added in the staining (FL-4). Gates were combined and 50,000 events were collected for each condition.

### Statistical evaluation

Statistical analyses were performed with the student T test. P values lower than 0.05 were considered significant. Plotted data are displayed as mean with standard deviation (SD).

## Results

### CD8^+ ^and CD4^+ ^T cells of the same donor recognize different EBV latent antigens

**Figure 1:**
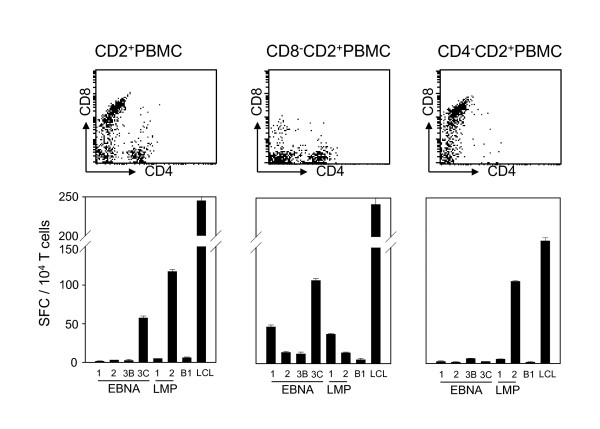
**CD4**^**+ **^**and CD8**^**+ **^**T cells of an HLA-B8**^**+ **^**donor recognize different EBV latent antigens**. Unseparated, CD8 or CD4 depleted, T cell enriched PBMCs were stimulated for two weeks with autologous LCL. Afterwards the cultures were tested for their CD4/CD8 content by flow cytometry (top row). Their antigen specificity was tested using autologous dendritic cells infected with recombinant vaccinia viruses encoding EBNA1, EBNA2, EBNA3B, EBNA3C, LMP1, LMP2 and BMLF1 (B1) in IFN-γ ELISPOT assays. As a positive control reactivity against the autologous LCL (LCL) was measured. 10^4 ^expanded T cells were added per ELISPOT well. One representative of three experiments is shown.

Autologous B lymphocyte cell lines (LCL) from a healthy HLA-B8^+ ^EBV carrier were used to stimulate unseparated and CD8 or CD4 depleted PBMCs for 2 weeks. Afterwards, the cultures were assayed for IFN-γ secretion in response to the autologous LCL or alternatively, autologous DCs infected with recombinant vaccinia viruses encoding the latent EBV antigens EBNA1, EBNA2, EBNA3B, EBNA3C, LMP1 and LMP2 as well as the lytic EBV antigen BMLF1 (Figure 1). The CD8^- ^T cell response was directed mainly towards EBNA1, EBNA3C and LMP1, while the CD4^- ^T cell response was predominantly LMP2 specific. The T cell-enriched PBMC fraction demonstrated the reactivities that dominated the CD4^+ ^and CD8^+ ^T cell responses, i.e., EBNA3C and LMP2, respectively. This donor is representative of published findings on immune responses to those EBV latent antigens that are expressed in spontaneously arising tumors like Hodgkin's lymphoma and nasopharyngeal carcinoma; EBNA1 and LMP1 are mainly recognized by CD4^+ ^T cells while LMP2 can be targeted by CD8^+ ^T cells at least in HLA-A2 positive individuals with considerable frequency[7].

### Characterization of a novel LMP2 derived HLA-B8 restricted CD8^+ ^T cell epitope

Four programs were used to predict HLA-B8 presented epitopes from LMP2 (Table 1). HLA-B8 was chosen since strong EBNA3A specific CD8^+ ^T cell responses restricted by this haplotype had been described[25], and we wanted to determine if the presence of strong CD8^+ ^T cell responses against the dominant EBNA3 latent antigens, would affect the occurrence of LMP2 specific CD8^+ ^T cells. Furthermore, HLA-B8 is the third most frequent HLA-B allele in the United States (8.5% of the population, after HLA-B44: 11.8% and HLA-B7: 10.9%)[26]. Two of these programs, SYFPEITHI[27] and BIMAS[28], predict peptide similarity to naturally eluted MHC ligands and estimate half time of disassociation from a particular MHC molecule, respectively. The other two programs, PAProC[29,30] and NetChop[31], were used to predict generation of the C-terminus of the candidate peptides by the proteasome. While N-terminal trimming can still occur in the ER, the C-terminus of most MHC class I ligands has to be generated by the proteasome[32]. PAProC version I bases its predictions on the in vitro protein digestions with the proteasome[29,30], while version II takes also in account the C-termini of naturally eluted MHC ligands[33]. These four programs were used to predict LMP2 derived octamers and nonamers for HLA-B8 presentation, since natural HLA-B8 ligands are generally of this length[27]. As summarized in Table 1, 6 peptides were predicted from the B95.8 LMP2 primary sequence to bind to HLA-B8 and to be generated by proteasomal C-terminal cleavage by at least one of the programs: 172-179, 234-242, 345-352, 347-355, 350-357 and 352-360.

**Table 1:** Predicted HLA-B8 binding peptides derived from LMP2.

LMP2 sequence	Amino acid sequence	SYFPEITHI rank/score*	BIMAS rank/value*	PAProC C-terminal cleavage prediction by version I or II	NetChop C-terminal cleavage prediction by version 2.0
172-179	**AAAQRKLL**	3^8^/22	1^8^/16.0	+/+	-

234-242	AYRRRWRRL	1^9^/24	2^9^/8.0	+/+	+

345-352	CPLSKILL	1^8^/26	2^8^/16.0	-/+	-

347-355	LSKILLARL	3^9^/20	3^9^/4.0	+/+	+

350-357	ILLARLFL	2^8^/25	3^8^/8.0	+/-	-

352-360	LARLFLYAL	2^9^/22	1^9^/16.0	-/+	+

* Separate predictions were performed for octamers (X^8^) and nonamers (X^9^). Therefore, each rank can appear twice. Predicted scores or values for the two MHC ligand prediction programs SYFPEITHI and BIMAS as well as the proteasomal cleavage prediction programs PAProC and NetChop are shown.

### LMP2_345-352 _is frequently recognized by HLA-B8^+ ^CD8^+ ^T cells

All predicted LMP2 peptides were synthetized and pulsed on autologous DCs for stimulation of HLA-B8^+ ^CD8^+ ^T cells. As a positive control, the dominant latent EBV epitope restricted by HLA-B8, EBNA3A_325-333_, was used. For a negative control, stimulation with unpulsed DCs was performed in parallel. After one week, the cultures were analyzed for IFN-γ secretion in response to autologous DCs infected with recombinant vaccinia viruses encoding LMP2 or EBNA3A or the control virus vvTK^- ^(Table 2). We used ELISPOT assays to enumerate IFN-γ secreting cells. Of the predicted LMP2 peptides, only LMP2_345-352 _stimulated LMP2 specific CD8^+ ^T cells in a large proportion of the HLA-B8^+ ^donors. By the criterion of 10 spots over background and at least a 1.5 ratio of vvLMP2 to vvTK^-^directed spots for a specific response, we could stimulate LMP2 specific CD8^+ ^T cells in 8 out of 11 donors (73%) using LMP2_345-352 _pulsed DCs. In the positive control stimulations, EBNA3A specific CD8^+ ^T cells could be detected in 9 of 11 donors (82%). After stimulation, the frequency of LMP2 specific CD8^+ ^T cells ranged between 19-581/5 × 10^4 ^cells. Similarly, 24-205/5 × 10^4 ^EBNA3A specific CD8^+ ^T cells could be obtained. These frequencies represent only two- to threefold expansion from the specific T cell numbers detected in fresh blood. This probably represents the fragility of purified CD8^+ ^T cell populations that are difficult to maintain in the absence of cytokines and/or CD4^+ ^T cells

**Table 2:** CD8^+ ^T lymphocytes from HLA-B8^+ ^donors frequently recognize the latent membrane protein 2 of Epstein Barr virus.

Donor	DCs only		**DC + LMP2**_**345-352**_		**DC + EBNA3A**_**325-333**_	
	**vvTK-**	**vvLMP2**	**vvTK-**	**vvLMP2**	**vvTK-**	**vvEBNA3A**

**#1**	0 ± 0*	0 ± 0	0 ± 0	**27.5 ± 1.5**	10 ± 7	**27 ± 0**

**#2**	12 ± 1	8.5 ± 1.5	7.5 ± 0.5	**19 ± 3**	17 ± 0	**30 ± 5**

**#3**	14 ± 1	0.5 ± 0.5	1 ± 0	**27.5 ± 3.5**	7.5 ± 0.5	**24 ± 3**

**#4**	105 ± 15	37.5 ± 2.5	25.5 ± 9.5	**70 ± 6**	26 ± 1	**79 ± 11**

**#5**	8.5 ± 0.5	6 ± 3	1.5 ± 1.5	10.5 ± 0.5	9 ± 1	13 ± 1

**#6**	232.5 ± 8.5	230.5 ± 3.5	305 ± 27	**581.5 ± 58.5**	115 ± 5	**205 ± 24**

**#7**	5 ± 3	5.5 ± 2.5	3.5 ± 1.5	**16 ± 2**	10 ± 1	19 ± 1

**#8**	1 ± 1	2 ± 1	0 ± 0	6 ± 1	7 ± 1	**60 ± 5**

**#9**	27.5 ± 3.5	5 ± 1	3.5 ± 0.5	10.5 ± 2.5	37.5 ± 7.5	**52 ± 2**

**#10**	13 ± 2	27 ± 7	14 ± 1	**74 ± 9**	15 ± 3	**68 ± 7**

**#11**	25 ± 11	28 ± 6	25 ± 5	**85 ± 21**	23 ± 2	**121.5 ± 13.5**

* spot forming cells (SFC)/5 × 10^4 ^cells. HLA-B8^+ ^CD8^+ ^T cells were stimulated for one week with dendritic cells pulsed with no peptide, LMP2_345-352 _or EBNA3A_325-333 _and then analyzed in ELISPOT assays against vvTK^-^, vvLMP2 and vvEBNA3A infected DCs. Specific responses as judged by the criterion of 10 spots over background and at least a 1.5 ratio of vvLMP2 to vvTK^-^stimulated spots are highlighted in bold.

The LMP2_345-352 _peptide, CPLSKILL, was predicted to be the best HLA-B8 binder by the SYFPEITHI program (score 26) and in the top group of three peptides predicted to be equally well retained by HLA-B8 in the BIMAS analysis (t1/2 = 16.0 min)(Table 1). Only PAProC version II predicted, however, that the proteasome can create the C-terminus of this peptide. Since this version also accommodates information about the termini of the natural HLA ligands it might not exclusively represent proteasomal specificity.

With the robust recognition of LMP2_345-352 _in the majority of HLA-B8^+ ^healthy EBV carriers, we next addressed the frequency of these CD8^+ ^T cells in peripheral blood.

### LMP2_345-352 _is recognized less frequently than EBNA3A_325-333 _in whole blood

**Figure 2:**
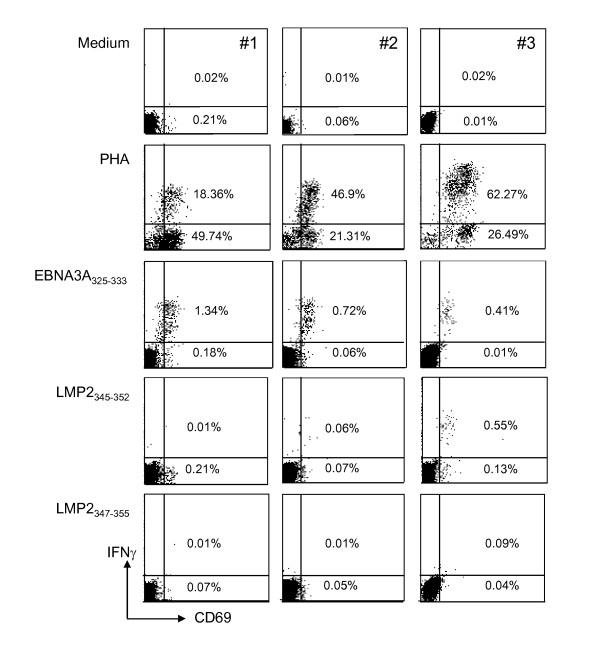
**Frequency of LMP2**_**345-352 **_**and EBNA3A**_**325-333 **_**specific CD8**^**+ **^**T cells in whole blood**. Whole blood of three representative of six HLA-B8 positive donors was stimulated with medium, PHA or the EBNA3A_325-333_, LMP2_345-352_, LMP2_347-355 _peptides. CD69 up-regulation and intracellular IFN-γ production was measured by flow cytometry after gating on living CD8^+ ^T cells. The indicated percentages represent CD69^+^/IFN-γ^+ ^(upper number) and CD69^+^/INF-γ^- ^(lower number) populations.

IFN-γ production by CD8^+ ^T cells in whole blood directly ex vivo was monitored after stimulation with EBNA3A_325-333 _and LMP2_345-352 _(Figure 2). As a positive control PHA and as negative controls medium and LMP2_347-355 _stimulations were used. Responses in three representative donors are shown in Figure 2. In donors with very strong EBNA3A_325-333 _specific responses like #1, LMP2_345-352 _specific IFN-γ producing CD8^+ ^T cells were undetectable. In other donors, there was a tendency that the strength of EBNA3A and LMP2 specific responses were inversely correlated. In 5/6 donors LMP2_345-352 _specific CD8^+ ^T cells responses were of lower frequency than EBNA3A_325-333 _specific responses. However, in 4/6 donors (67%) LMP2_345-352 _specific CD8^+ ^T cells responses were detectable ranging from 0.1 to 0.68%. 0.55% CD69^+^IFN-γ^+ ^plus 0.13% CD69^+^IFN-γ^- ^of peripheral CD8^+ ^T cells was the highest frequency detected for LMP2_345-352_.

### In vitro stimulation with peptide pulsed DCs can expand LMP2_426-434_, LMP2_345-352 _and EBNA3A_325-333 _specific CD8^+ ^T cell responses to high levels

**Figure 3:**
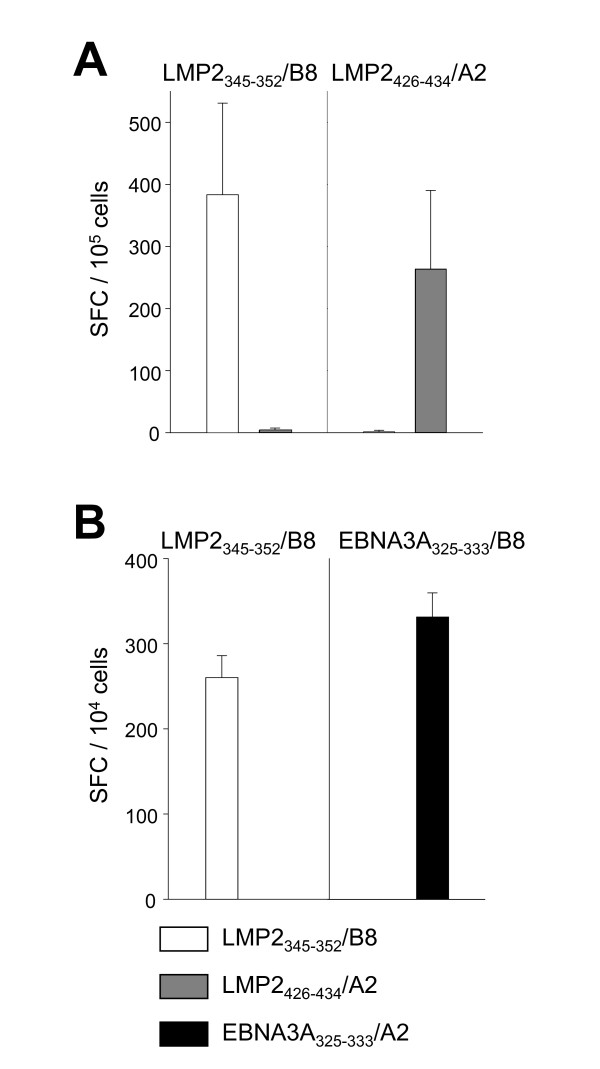
**Comparison of LMP2**_**345-352 **_**frequency with EBNA3A**_**325-333 **_**and LMP2**_**426-434 **_**in HLA-B8**^**+**^**/-A2**^**+ **^**and HLA-B8**^**+**^**/-A2**^- ^**donors after in vitro stimulation**. IFN-γ ELISPOT assays were performed with CD8^+ ^T cell cultures expanded for 1 week with peptide pulsed DCs (stimulatory peptides are indicated above panels) and restimulation for IFN-γ secretion by direct addition of 10 μM peptide (readout peptides are indicated in the legend). In HLA-A2^+^/-B8^+ ^donors, the LMP2_426-434 _and LMP2_345-352 _specific responses were compared (A). In HLA-B8^+^/-A2^- ^donors, the EBNA3A_325-333 _and LMP2_345-352 _specific responses were addressed (B). One representative of three donors is shown in (A) and in (B). 10^5 ^and 10^4 ^expanded T cells were added per ELISPOT well in (A) and (B), respectively.

HLA-B8^+^/-A2^+ ^and HLA-B8^+^/-A2^- ^CD8^+ ^T cells were stimulated for one week with LMP2_426-434_, LMP2_345-352 _and EBNA3A_325-333 _peptide pulsed autologous DCs. After one week the cultures were assayed for IFN-γ secretion in ELISPOT by addition of the peptides (Figure 3). Figure 3A is representative for 3 HLA-A2^+^/-B8^+ ^donors. In all donors LMP2_345-352 _and LMP2_426-434 _specific CD8^+ ^T cells could be expanded to similar levels. Figure 3B is representative for three HLA-A2^-^/-B8^+ ^donors whose LMP2_345-352 _and EBNA3A_325-333 _CD8^+ ^T cells could be expanded to similar levels by peptide pulsed DCs. These donors were tested in addition to the donors described in Table 2.

### LMP2_345-352 _and EBNA3A_325-333 _specific CD8^+ ^T cell clones uniformly up-regulate CD69 upon peptide encounter

**Figure 4:**
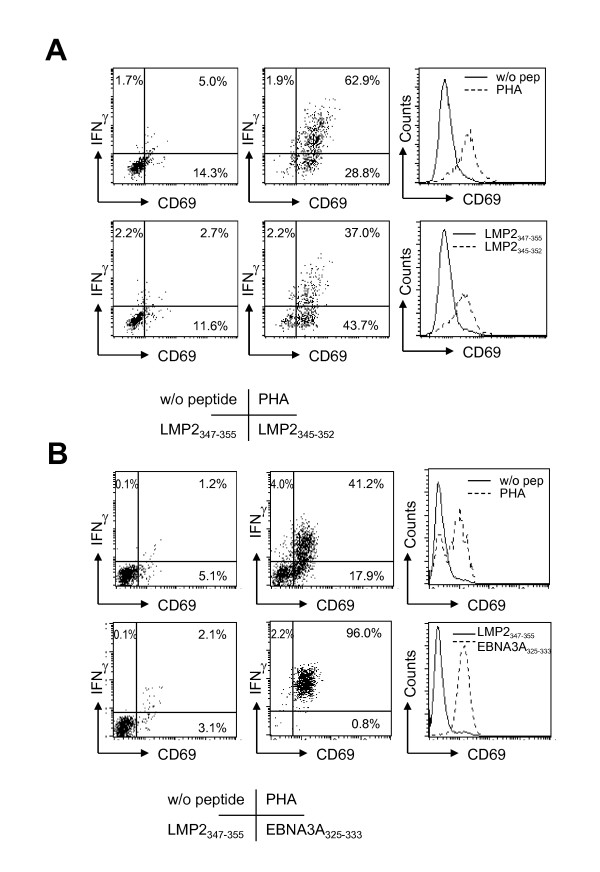
**CD69 up-regulation and IFN-γ secretion by LMP2 and EBNA3A specific HLA-B8 restricted CD8**^**+ **^**T cell clones**. Clonal T cells were stimulated with medium, PHA, the control LMP2_347-355 _peptide or the cognate LMP2_345-352 _or EBNA3A_325-333 _peptides. CD69 surface expression and intracellular IFN-γ was analyzed. Dot blot and histogram analysis was performed after gating on CD8^+ ^T cells. The LMP2_345-352 _specific CD8^+ ^T cell clone #3 (A) and the EBNA3A_325-333 _specific CD8^+ ^T cell clone MS.B11 (B) were analyzed. The presented data are representative for five LMP2_345-352 _specific CD8^+ ^T cell clones.

Five LMP2_345-352 _specific CD8^+ ^T cell clones, #1-5, were generated from the 0.3 cells/well dilution of a CD8^+ ^T cell line with >1% frequency for this specificity. Upon addition of 10 μl 1 mM LMP2_345-352 _peptide solution (20 μM final concentration) in combination with the co-stimulatory antibodies CD49d and CD28, these clones uniformly up-regulate CD69, while only 30-50% produced IFN-γ. Both parameters were measured after intracellular IFN-γ staining and CD69 staining using flow cytometry. Similarly upon PHA stimulation, 30-70% produce IFN-γ, while nearly all clonal cells up-regulate CD69. As negative controls, medium only or the control peptide LMP2_347-355 _were used (Figure 4A). For comparison within different antigen specificities, we subjected the EBNA3A_325-333 _specific HLA-B8 restricted MS.B11 CD8^+ ^T cell clone, generated by the same cloning strategy, to a similar analysis (Figure 4B). Again, CD69 was up-regulated on 65-78% of PHA stimulated clonal T cells, while IFN-γ production was induced in 38-41%. In response to the cognate EBNA3A_325-333 _peptide, however, both CD69 as well as IFN-γ were induced in more that 95% of MS.B11 CD8^+ ^T cells. Medium and the non-cognate LMP2_347-355 _peptide served as negative controls. The homogeneity of CD69 up-regulation upon cognate peptide encounter argues that the obtained T cell populations, LMP2_345-352 _specific clones #1-5 and the EBNA3A_325-333 _specific MS.B11 clone, are indeed of singular antigen specificity. Since they were derived from 0.3 cells/well cloning plates with around 10 expanding wells per plate, these T cell populations can be considered clonal.

Together with the CD69/IFN-γ pattern observed in whole blood these findings indicate that determinations of antigen specific CD8^+ ^T cell frequencies should take into account IFN-γ^+^/CD69^+ ^as well as IFN-γ^-^/CD69^+ ^T cells. This also holds true for EBNA1 specific CD4^+ ^T cell clones (data not shown). Namely, after stimulation with epitope pulsed HLA matched lymphoma cells only a proportion (up to 70%) of CD69^+ ^cells in an EBNA1 specific Th1 CD4^+ ^T cell clone can be visualized by intracellular IFN-γ staining. Therefore, CD69 is not only suitable to co-stain and thereby better visualize IFN-γ secreting cells, but can detect additional antigen specific CTL that might appear IFN-γ negative at a given time-point, possibly due to lack of synchronization in their antigen response.

### LMP2_345-352 _specific CD8^+ ^T cell clones recognize HLA-B8 matched LCL

**Figure 5:**
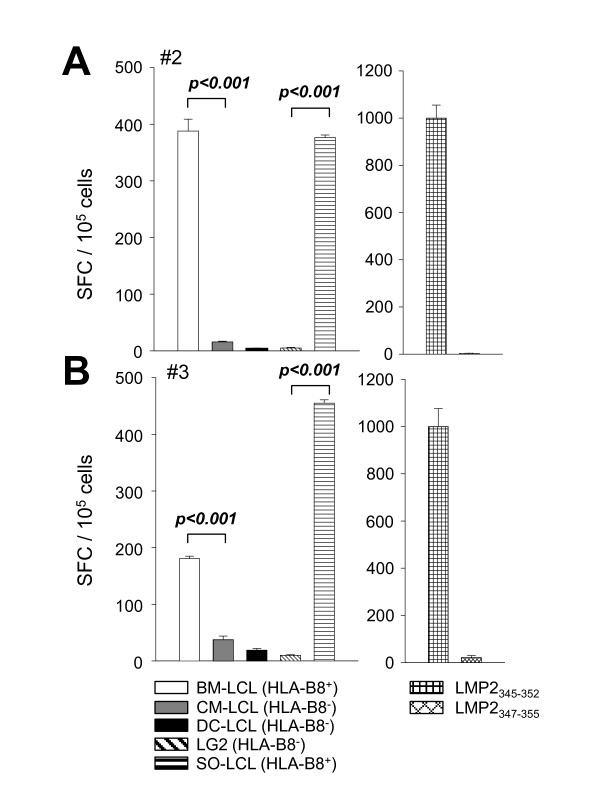
**Recognition of LCL by LMP2**_**345-352 **_**specific T cell clones**. The LMP2_345-352 _specific CD8^+ ^T cell clones #2 (A) and #3 (B) were stimulated with the BM-LCL (HLA-B8^+^), CM-LCL (HLA-B8^-^), DC-LCL (HLA-B8^-^), LG2 (HLA-B8^-^) and SO-LCL (HLA-B8^+^) as well as direct addition of the LMP2_345-352 _and LMP2_347-355 _peptides in IFN-γ ELISPOT assays. Representative data for two out of five LMP2_345-352 _specific CD8^+ ^T cell clones are shown. 10^5 ^clonal T cells were added per ELISPOT well.

The LMP2_345-352 _specific CD8^+ ^T cell clones were tested for recognition of HLA-B8 matched LCL in IFN-γ ELISPOT assays (Figure 5). The HLA-B8^+ ^cell lines BM-LCL and SO-LCL were recognized by clones #2 and #3, while the HLA-B8^- ^lines CM-LCL, DC-LCL and LG2 stimulated no IFN-γ secretion. In comparison to direct addition of 5 μl 1 mM LMP2_345-352 _peptide solution to the ELISPOT wells (25 μM final concentration), the response to the HLA matched LCLs was significantly lower. As a negative control, the same amount of the LMP2_347-355 _peptide was added.

### LMP2_345-352 _specific CD8^+ ^T cell clones are HLA-B8 restricted and recognize peptide concentrations down to 10 nM

**Figure 6:**
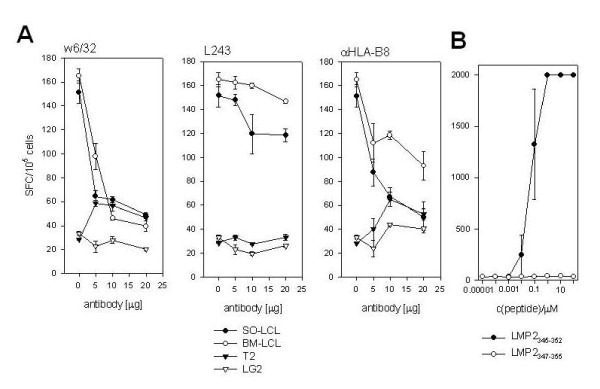
**Peptide affinity and HLA restriction by LMP2**_**345-352 **_**specific T cell clone #3**. IFN-γ ELISPOT assays were performed with HLA-B8^+ ^SO-LCL and BM-LCL as well as HLA-B8^- ^LG2 and T2 cell lines as stimulators for the LMP2 specific T cell clone #3 in the presence of titrated doses of the αHLA class I antibody w6/32, the αHLA-DR antibody L243 and the αHLA-B8 antibody (A). Peptide affinity of clone #3 was determined by direct addition of increasing concentrations of the LMP2_345-352 _and the LMP2_347-355 _peptides to clonal T cells in INFγ ELISPOT assays (B). Data for one representative of five LMP2_345-352 _specific CD8^+ ^T cell clones are shown. 10^5 ^clonal T cells were added per ELISPOT well.

IFN-γ secretion in response to HLA-B8^+ ^and HLA-B8^- ^LCL was performed in the presence of increasing antibody concentrations (Figure 6A). Three antibodies were used: the HLA class I specific antibody w6/32, the HLA-DR specific antibody L243 and an HLA-B8 specific IgM antibody. Recognition of HLA-B8^+ ^SO-LCL and BM-LCL by the LMP2_345-352 _specific CD8^+ ^T cell clones was inhibited by the w6/32 and αHLA-B8 antibodies, while L243 had only minimal effects on the recognition (example shown for clone #3 in Figure 3A). The HLA-B8^- ^cell lines T2 and LG2 served as negative controls.

In peptide titration experiments, recognition of the LMP2_345-352 _epitope was still detectable at a concentration of 10 nM (Figure 6B). Half maximal recognition is difficult to determine in ELISPOT assays since cellular contact becomes limiting at low cell numbers and spot evaluation is difficult at high cell numbers per well.

### LMP2_345-352 _specific CD8^+ ^T cell clones kill peptide pulsed targets and HLA-B8^+ ^LCL

**Figure 7:**
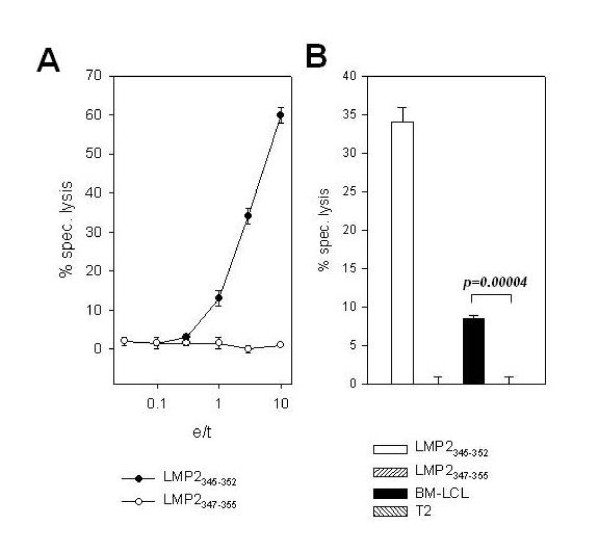
**Cytolytic activity of LMP2**_**345-352 **_**specific T cell clone #3**. ^51^Cr release assays were performed with LMP2_345-352 _specific T cell clone #3 and peptide pulsed DCs (A) and HLA-B8^+ ^BM-LCL and HLA-B8^- ^T 2 cells at an e/t ratio of 3:1 (B). Results for one representative of five LMP2_345-352 _specific CD8^+ ^T cell clones are shown.

^51^Cr release assays were performed using peptide pulsed DCs or LCLs as targets and LMP2_345-352 _specific CD8^+ ^T cell clones as effectors (Figure 7). Figure 7A shows that LMP2_345-352 _specific T cell clones selectively killed DCs pulsed with the cognate, but not with the non-cognate peptide. At an e/t ratio of 3:1, 30% specific lysis against LMP2_345-352 _peptide pulsed DCs was obtained, while only 10% of the HLA-B8^+ ^BM-LCL cells were killed (Figure 7B). The background lysis for HLA-B8^- ^T2 cells and LMP2_347-355 _pulsed DCs was always below 5%. Therefore, killing of HLA-B8 matched LCL was 3 times lower than that of peptide pulsed targets. This corresponds to the reduced recognition of HLA-B8^+ ^LCL in comparison to peptide addition in the ELISPOT assays and probably represents limiting epitope density on the LCL[34,35]. Similar cytolytic behavior was observed for all five LMP2_345-352 _specific CD8^+ ^T cell clones. Thus, the low cytotoxicity of LMP2_345-352 _specific CD8^+ ^T cells against autologous LCLs, although detectable in our in vitro assays, might have limited protective value in the immune control of virally transformed B cells in vivo.

### LMP2_345-352 _specific CD8^+ ^T cell clones recognize LMP2 expressing Hodgkin's lymphoma cells

**Figure 8:**
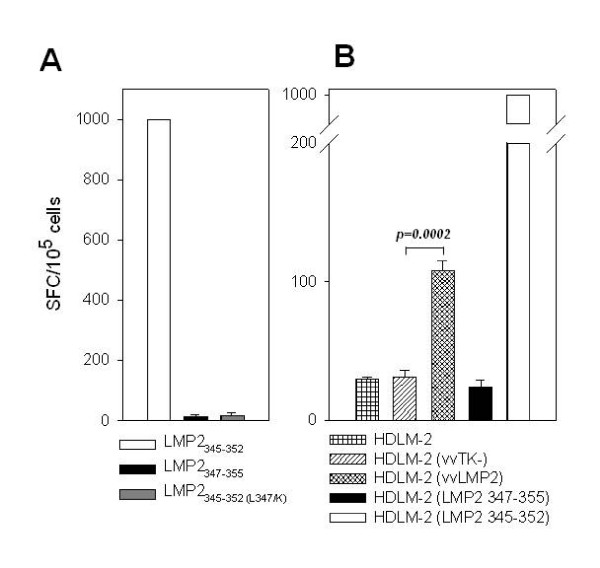
**Recognition of Hodgkin's lymphoma cells by LMP2**_**345-352 **_**specific T cell clone #3**. A: Substitution of position 3 in the cognate LMP2_345-352 _peptide with the favored HLA-B8 anchor amino acid K leads to abolishment of the recognition by LMP2_345-352 _specific CD8^+ ^T cell clones as exemplified by clone #3. The indicated peptides were added to the clonal T cells. B: HLA-B8^+ ^EBV^- ^Hodgkin's lymphoma cells were either left unmanipulated, peptide pulsed, infected with recombinant vaccinia virus encoding LMP2 (vvLMP2) or vaccinia control virus (vvTK-) for stimulation of the LMP2_345-352 _specific CD8^+ ^T cell clone #3 in IFNγ ELISPOT assays. Data for one representative of five LMP2_345-352 _specific CD8^+ ^T cell clones are shown. 10^5 ^clonal T cells were added per ELISPOT well.

In order to improve CD8^+ ^T cell recognition by improved binding of the HLA-B8 restricted LMP2 epitope, we investigated the heteroclitic peptide CPKSKILL in which relative position 3, LMP2 amino acid 347, was changed from L to K according to the optimal HLA-B8 binding motif XXKXKXXL[27]. Unfortunately, this change abolished the recognition by our LMP2_345-352 _specific CD8^+ ^T cell clones upon direct addition of the peptide into the ELISPOT assay (Figure 8A). The unchanged epitope and the LMP2_347-355 _peptide were used as positive and negative controls, respectively.

Endogenous expression of LMP2 by recombinant vaccinia infection (vvLMP2) rendered EBV^- ^HLA-B8^+ ^Hodgkin's cells recognizable for the LMP2_345-352 _specific CD8^+ ^T cell clones (Figure 8B). Control vaccinia infected, untreated and control peptide pulsed HDLM-2 cells did not cause IFN-γ secretion. LMP2_345-352 _peptide pulsing, however, stimulated a stronger response than vvLMP2 infection. This can probably be explained by the low infection efficiency (10%) of our recombinant vaccinia viruses for B cell lines like Hodgkin's lymphoma, Burkitt's lymphoma and LCL (data not shown). The observed response against vvLMP2 infected HDLM-2 cells suggests that LMP2 gets processed onto HLA-B8 in the infected cells and therefore endogenously LMP2 expressing Hodgkin's lymphoma cells can be targeted by the LMP2_345-352 _specific CD8^+ ^T cell response.

## Discussion

In the present work we describe responses to the latent EBV antigen LMP2, which is expressed in Hodgkin's lymphoma and nasopharyngeal carcinoma. We studied HLA-B8 restricted CD8^+ ^T cell responses directed against this antigen and demonstrate that LMP2 reactivity is present despite the strong EBNA3A specific CD8^+ ^T cell responses that are consistently associated with this HLA allele (this study and [25]). Furthermore, we determined the new HLA-B8 restricted epitope CPLSKILL (LMP2_345-352_), recognized by LMP2 specific CD8^+ ^T cells. Although the frequency in whole peripheral blood is usually lower for LMP2 specific than for EBNA3A specific HLA-B8 restricted CD8^+ ^T cells, both reactivities can be expanded to similar levels upon in vitro stimulation with peptide pulsed DCs. The LMP2_345-352 _specific CD8^+ ^T cell clones that we isolated could recognize endogenously expressed LMP2 of HLA-B8^+ ^LCL and Hodgkin's lymphoma cells, leading to IFN-γ secretion and cytotoxicity.

Our data suggest that even in HLA haplotypes like HLA-B8 that give rise to strong CD8^+ ^T cell responses against the EBNA3 antigens, LMP2 specific CD8^+ ^T cell responses are present. However, unlike the EBNA3 antigens, LMP2 is also expressed in EBV associated malignancies like Hodgkin's lymphoma and nasopharyngeal carcinoma that have lost EBNA3 expression[4]. Thus, induction and maintenance of EBV specific T cells against antigens present in these tumors might be necessary to restrict transition from infected centroblast and -cytes with the same EBV antigen expression to Hodgkin's lymphoma. Indeed selective loss of individual T cell specificities against EBV antigens have been found in patients with Hodgkin's lymphoma, Burkitt's lymphoma and AIDS associated EBV positive lymphoma[36-38]. In these instances loss of EBNA1 specific T cell reactivity was found. This selective loss might favor the escape of EBV associated tumors with restricted antigen expression, and LMP2 specific T cell responses should also be investigated along these lines.

In the case of nasopharyngeal carcinoma, an epithelial tumor that is to 100% associated with EBV, the EBV latent antigen expression might even be further restricted to mainly EBNA1 and LMP2. EBNA1 and LMP2 mRNA are easily detected by RT-PCR in samples of this tumor, while LMP1 message is difficult to amplify. LMP1 protein is even undetectable in up to 65% of NPC biopsies[39,40]. Moreover, NPC patients are the only EBV carriers that reproducibly develop LMP2 specific antibody responses[41,42], indicating a significant level of LMP2 protein expression. The etiology of NPC is still unclear. However, systemic loss of T cell responses against distinct EBV antigens could so far not be documented, even so individual T cell specificities restricted by certain HLA alleles were found to be compromised[43]. Instead, suppression of these systemic T cell responses in the local tumor microenvironment have been suggested[44,45]. Therefore, EBV associated lymphomas might escape immune control in part due to systemic loss of distinct EBV antigen specific T cell responses, while nasopharyngeal carcinoma might develop in the presence of unaltered systemic EBV specific T cell responses due to an immune suppressive tumor microenvironment[46].

The efficient expansion of LMP2_345-352 _specific CD8^+ ^T cells from low starting frequencies with peptide-pulsed DCs suggests that DCs could be used to expand EBV latency II specific CD8^+ ^T cells. For dominant HLA class I restricted EBV derived CD8^+ ^T cell epitopes this has been reported previously[47-49]. Here we extend this finding to demonstrate that initially subdominant CD8^+ ^T cell responses can be boosted in vitro to similar levels as the dominant EBV responses using peptide pulsed DCs. These results are in line with efficient expansion of LMP2 specific T cells with antigen expressing DCs for adoptive transfer into Hodgkin's lymphoma patients[50]. Moreover, EBV antigen presenting DCs can also prime, and not only restimulate protective T cell responses[51,52]. Therefore, subdominant, but tumor associated EBV antigen expression in DCs should be explored as immunotherapy against spontaneously occurring EBV associated malignancies with reduced viral antigen expression.

In conclusion, our findings suggest that LMP2 specific CD8^+ ^T cells are primed and maintained even in HLA haplotypes that generate strong EBNA3 specific CD8^+ ^T cell responses. The LMP2 response might be necessary to prevent and treat EBV associated malignancies with reduced viral antigen expression like Hodgkin's lymphoma, inhibiting the outgrowth of transformed EBV^+ ^germinal center B cells that express this protein.

## Competing interests

The authors declare that they have no competing interests.

## Authors' contributions

MLT performed the experiments and wrote part of the manuscript. CM designed the study and wrote part of the manuscript. All authors read and approved the final manuscript.
